# Barriers to Receiving Applied Behavior Analysis Services in Children With Autism Spectrum Disorder

**DOI:** 10.7759/cureus.48585

**Published:** 2023-11-09

**Authors:** Emily R Littman, Leslie Gavin, Andrew Broda, Ansley C Hodges, Lisa Spector

**Affiliations:** 1 Medicine, University of Central Florida College of Medicine, Orlando, USA; 2 Pediatrics, Nemours Children's Hospital, Orlando, USA; 3 General Surgery, Anne Arundel Medical Center, Annapolis, USA; 4 Behavioral Health, Nemours Children's Hospital, Orlando, USA; 5 Developmental and Behavioral Pediatrics, Nemours Children's Hospital, Orlando, USA

**Keywords:** socioeconomic status, access to care, barriers, applied behavior analysis, autism spectrum disorder

## Abstract

Introduction

Applied behavior analysis (ABA) is commonly used to treat children with autism spectrum disorder (ASD). The objective of this study is to evaluate barriers to ABA treatment in ASD.

Methods

A voluntary 51-question survey, including demographics, socioeconomic status, parental assertiveness/self-advocacy, and parent perceptions, was provided to caregivers of children aged one to eight years old diagnosed with ASD. The survey consisted of a series of yes/no, five-point Likert scale, multiple-choice, and text field questions.

Results

A total of 540 surveys were completed. The median time since ASD diagnosis was three to five years ago. Respondents were identified as receiving ABA (r-ABA) vs. not receiving ABA (n-ABA). Respondents were from Florida (60%; r-ABA = 61.7%; n-ABA = 57.0%), Pennsylvania (18%; r-ABA = 21.3%; n-ABA = 12.8%), Delaware (17%; r-ABA = 11.7%; n-ABA = 26.8%), and New Jersey (5%; r-ABA = 5.3%; n-ABA = 3.4%) (p < 0.001). Caregiver belief in ABA treatment, empowerment, and comfort level were greater for r-ABA (4.20 ± 0.72; 3.19 ± 0.93; 4.35 ± 0.72) compared to n-ABA (4.06 ± 0.83; 2.90 ± 1.00; 4.03 ± 0.91), respectively (mean ± SD).

Conclusion

Barriers to accessing ABA services are a multifactorial issue. Location, income, education of the caregiver, time since ASD diagnosis for the child, as well as caregiver empowerment, belief in treatment, and level of comfort in accessing services most likely contribute to children with ASD not receiving ABA. Empowering parents on their impact on their child’s treatment may improve ABA use.

## Introduction

Autism spectrum disorder (ASD) is a neurodevelopmental disorder that has a prevalence of one in 36 in the United States and is characterized by social communication deficits and repetitive sensory and motor behaviors [1-4]. Diagnosis is challenging and is often not made until age four; prompt diagnosis and therapy are essential in promoting optimal outcomes. Diagnostic barriers have been identified previously, including those with low socioeconomic status (SES) and members of minority groups, such as African Americans, who are diagnosed with ASD 1.5 years later than their peers [5-7].

It is well known that children with ASD who receive early diagnosis and intervention are more likely to have long-term positive outcomes [8,9]. Children receiving intervention before age five are more likely to successfully integrate in normal classroom settings and perform well [10]. Children receiving long-term applied behavior analysis (ABA) therapy experienced 47% improvement in intellectual function [11,12]. In addition to its effects on children, families also experience caregiver strain, emotional distress, financial burden, and higher divorce rates [13-15]. While approaches for ASD treatment may vary, ABA is the most common treatment due to abundant evidence of efficacy [13].

ABA utilizes one-on-one interactions with board-certified behavior analysts (BCBAs) and group-oriented approaches to build social and cognitive skills, eliminate negative behaviors, improve motor skills, and enhance quality of life [12,13,16]. For children with ASD, an intensive ABA regimen, occurring anywhere from five to seven days a week for 40 hours weekly, is recommended [3,11].

Despite growing evidence demonstrating benefit, a number of barriers, including cost, lack of time, and limited ABA access, exist [11-13,16,17]. Knowledge deficits and societal stigma also impede access to quality services [18,19]. Many times parents are unaware of the developmental progress made possible with ABA [20]. Research is still needed to understand why children with ASD do not receive prompt ABA following diagnosis. The purpose of this study is to identify the factors that prevent children from receiving these services.

## Materials and methods

Design

This is a single-center, cross-sectional, descriptive study utilizing a convenience sample of subjects who responded to a request to participate in a survey.

Setting

Four Nemours Children’s Health locations participated in this study: Florida, Delaware, New Jersey, and Pennsylvania. Institutional Review Board approval was obtained through Nemours Children’s Hospital. As this involved survey data collection, we obtained a waiver of written consent since survey completion implied consent for data collection.

Inclusion/exclusion criteria

The survey was sent to caregivers if they had a child who had been diagnosed with ASD and had received care through Nemours Children’s Hospital, consented to receive surveys, and reported English as their primary language. Of survey respondents, children were included if they were between ages one and eight years old.

Measures

The survey was created and adapted from a variety of validated surveys in the field addressing socioeconomic status, parental assertiveness, self-advocacy, and parental perception. The survey was developed by a research team comprised of a developmental and behavioral pediatrician, a clinical child psychologist, a doctorally prepared BCBA, a parent of a child with ASD, and a medical student. A 51-item survey was developed that consisted of a series of yes/no, five-point Likert scale, multiple-choice, and text field questions. The questions addressed factors believed to contribute to caregivers of ASD children receiving ABA, including demographics, socioeconomic status, parental assertiveness/self-advocacy, and parent perceptions (Appendix).

Data collection

Study data were collected, managed, and stored using the secure, de-identified, electronic data capture tool, REDCap (Vanderbilt University, Nashville, TN), hosted at Nemours Children’s Health [21,22]. Eligible participants could complete the 20-minute survey at their leisure using a secure REDCap survey link, which expired 14 days after receipt.

Statistical analysis

Data output was generated from REDCap. Survey responses were collected predominantly as Likert scale questions and converted into dichotomous variables. Responses “Strongly Agree” and “Agree” were coded as “Yes” and “Disagree” and “Strongly Disagree” were coded as “No.” The survey response “Neutral” was excluded from the data analysis. Descriptive statistics were performed on caregiver demographics and responses to ABA barriers. Pearson’s chi-square test (χ2) and independent Student’s t-test were performed comparing those receiving ABA (r-ABA) and those who have never received ABA (n-ABA) on response variables as indicated by the variable type. Multivariate logistic regression analysis was performed on responses to ABA barriers comparing ABA utilization. The demographic variables of race, income, and insurance status were used as controls for potential co-variability. Statistical analysis was performed using IBM SPSS Statistics 28 (IBM Corp., Armonk, NY), SAS 9.4 (SAS Institute Inc., Cary, NC), and MathWorks MATLAB_R2020a (MathWorks, Natick, MA). Significance was defined as p < 0.050.

Factor analysis

Twelve questions relating to caregiver beliefs in treatment, empowerment, and comfort level were examined for factorability. Outliers were not an issue since all the questions existed on a scale from one to five. The Kaiser-Meyer-Olkin measure of sampling adequacy and Bartlett’s test of sphericity were used to see if the data were appropriate for factor analysis. The entire analysis was done using SAS 9.4.

## Results

Participant demographics

A total of 540 surveys were received, with 444 completed surveys, and 73 incomplete surveys. A total of 23 participants whose child was diagnosed within the last six months due to not having proper time to receive services were excluded. For each question, participants had the option of answering "other," "don't know," or to omit the question. Of the respondents, 92.5% were female and 73.1% were aged 30-44 years. Ethnicities of participants included 56% (n = 234) Caucasian, 13% (n = 55) African American, 4.8% (n = 20) Asian, and 17% (n = 73) Latino or Hispanic. If respondents selected multiple ethnicities, they were reclassified as "multiracial." No significant differences in caregiver age, marital status, and ethnicity between their child receiving ABA and not receiving ABA were noted (Table 1).

**Table 1:** Caregiver demographics based on ABA status * = Statistically significant difference between subgroups defined as p < 0.050. ABA: applied behavior analysis; VA: Veterans Affairs; GED: General Educational Diploma; PhD: Doctor of Philosophy; EdD: Doctor of Education; DVM: Doctor of Veterinary Medicine; MD: Doctor of Medicine; JD: Juris Doctor; DDS: Doctor of Dental Surgery; CHIP: Children's Health Insurance Program.

Variable	Have received ABA therapy, N (%)	Have never received ABA therapy, N (%)	p-value
Age of parent			0.445
18-29 years old	17 (6.3%)	12 (7.7%)	
30-44 years old	199 (74.3%)	111 (71.2%)	
45-59 years old	49 (18.3%)	28 (17.9%)	
60+ years old	3 (1.1%)	5 (3.2%)	
Marital status			0.280
Married or living with a partner	202 (75.7%)	106 (67.9%)	
Divorced/separated	27 (10.1%)	19 (12.2%)	
Widowed	3 (1.1%)	1 (0.6%)	
Single never married	35 (13.1%)	30 (19.2%)	
Ethnicity			0.489
Caucasian	147 (55.1%)	87 (56.5%)	
African American	33 (12.4%)	22 (14.3%)	
Latino or Hispanic	47 (17.6%)	26 (16.9%)	
Asian/Native Hawaiian/Pacific Islander	14 (5.2%)	6 (3.9%)	
Multiracial	20 (7.5%)	6 (3.9%)	
Other/unknown	2.2% (6)	4.5% (7)	
State of residence			<0.001*
Florida	174 (61.7%)	85 (57.0%)	
Delaware	33 (11.7%)	40 (26.8%)	
New Jersey	15 (5.3%)	5 (3.4%)	
Pennsylvania	60 (21.3%)	18 (12.8%)	
Length of time since diagnosis			0.067
7-12 months ago	22 (7.7%)	16 (10.1%)	
1-2 years ago	53 (18.6%)	43 (27.0%)	
3-5 years ago	119 (41.8%)	64 (40.3%)	
Greater than 5 years ago	91 (31.9%)	36 (22.6%)	
Income			0.001*
Less than $5,000	8 (3.2%)	7 (4.9%)	
$5,000-$24,999	24 (9.7%)	24 (16.9%)	
$25,000-$49,999	48 (19.4%)	38 (26.8%)	
$50,000-$74,999	35 (14.2%)	23 (16.2%)	
$75,000-$99,999	3 (17.4%)	14 (9.9%)	
$100,000-$149,999	44 (17.8%)	17 (12.0%)	
≥$150,000	45 (18.2%)	19 (13.4%)	
Education				0.003*
Didn't finish high school	1 (0.4%)	6 (3.9%)		
Didn't finish high school, but completed a technical/vocational program	0 (0.0%)	1 (0.6%)		
Completed high school and a technical/vocational program	16 (6.0%)	14 (9.1%)		
High school diploma or equivalency (GED)	22 (8.2%)	21 (13.6%)		
Less than 2 years of college	37 (13.9%)	17 (11.0%)		
Associate degree (junior college) or equivalent	38 (14.2%)	33 (21.4%)		
Bachelor's degree	83 (31.1%)	35 (22.7%)		
Master's degree or other post-graduate training	56 (21.0%)	19 (12.3%)		
Doctorate or other professional degree (PhD, EdD, DVM, MD, JD, DDS, etc.)	14 (5.2%)	8 (5.2%)		
Employment status			0.021*
Homemaking or raising children full-time	94 (35.3%)	44 (28.6%)	
Retired	2 (0.8%)	2 (1.3%)	
Disabled	6 (2.3%)	13 (8.4%)	
Unemployed or laid off	20 (5.3%)	6 (3.9%)	
Working part-time	25 (9.4%)	20 (13.0%)	
Working full-time	125 (47.0%)	69 (44.8%)	
Health insurance			0.654
Current or former employer or union	105 (37.0%)	55 (34.8%)	
Insurance purchased directly from an Insurance company	11 (3.9%)	4 (2.5%)	
TRICARE, VA, or other military health care	11 (3.9%)	4 (2.5%)	
Medicaid, Medical Assistance, CHIP, or any kind of government-assistance plan	120 (42.3%)	78 (49.4%)	
No health insurance or other	3 (1.1%)	3 (1.9%)	
Primary health insurance plus supplemental government assistance plan	34 (12.0%)	14 (8.9%)	

Location

Respondents were from Florida (r-ABA = 61.7%; n-ABA = 57.0%), Delaware (r-ABA = 11.7%; n-ABA = 26.8%), New Jersey (r-ABA = 5.3%; n-ABA = 3.4%), and Pennsylvania (r-ABA = 21.3%; n-ABA = 12.8%) (p < 0.001; Table 1).

Time since ASD diagnosis

**Figure 1:**
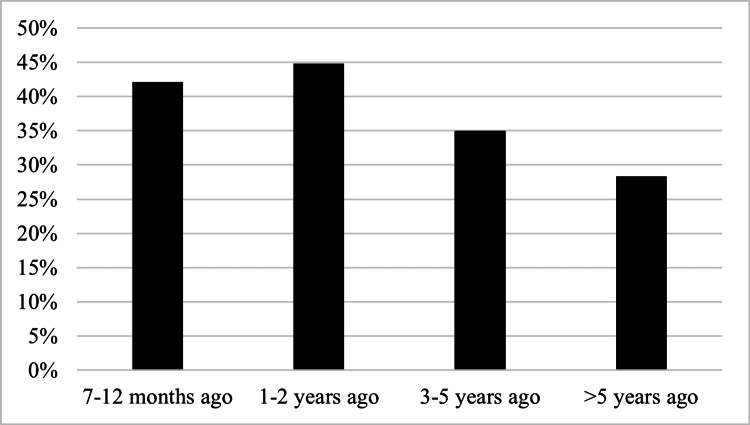
Length of time since ASD diagnosis of those who have never received ABA ASD: autism spectrum disorder; ABA: applied behavior analysis.

When comparing raw survey responses for time since diagnosis, there was no statistical significance among groups (p = 0.067). However, when subgroup analysis was performed, caregivers with r-ABA children were more likely to be diagnosed at least three years ago (r-ABA = 73.7%; n-ABA = 62.9%) (p = 0.018) than those diagnosed within the last two years (r-ABA = 26.3%; n-ABA = 37.1%) (Table 1 and Figure 1). When comparing r-ABA vs. n-ABA for each time since diagnosis, those who were more newly diagnosed were less likely to have received ABA, while those who had more time since their diagnosis were more likely to receive ABA.

Caregiver income

**Figure 2:**
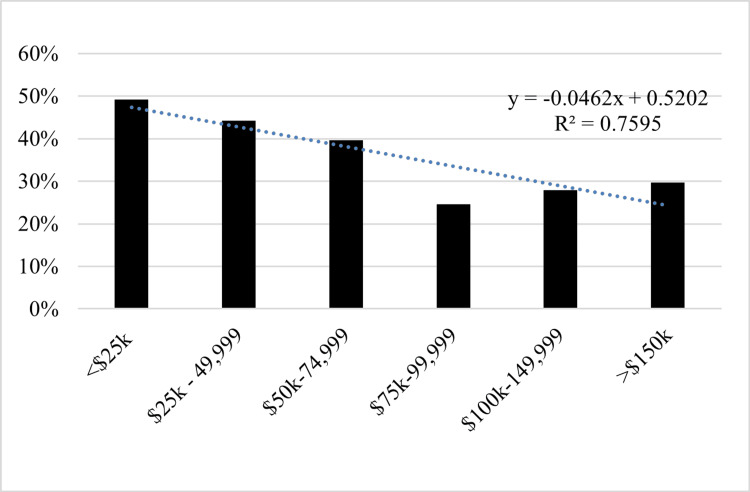
Income level of those who have never received ABA ABA: applied behavior analysis.

Participants who reported higher incomes and education levels were more likely to receive ABA. Participants reported a total annual household income below $50,000 (r-ABA = 32.3%; n-ABA = 48.6%), $50,000-$100,000 (r-ABA = 31.6%; n-ABA = 26.1%), and greater than $100,000 (r-ABA = 36.0%; n-ABA = 25.4%) (p = 0.001; Table 1 and Figure 2).

Education

**Figure 3:**
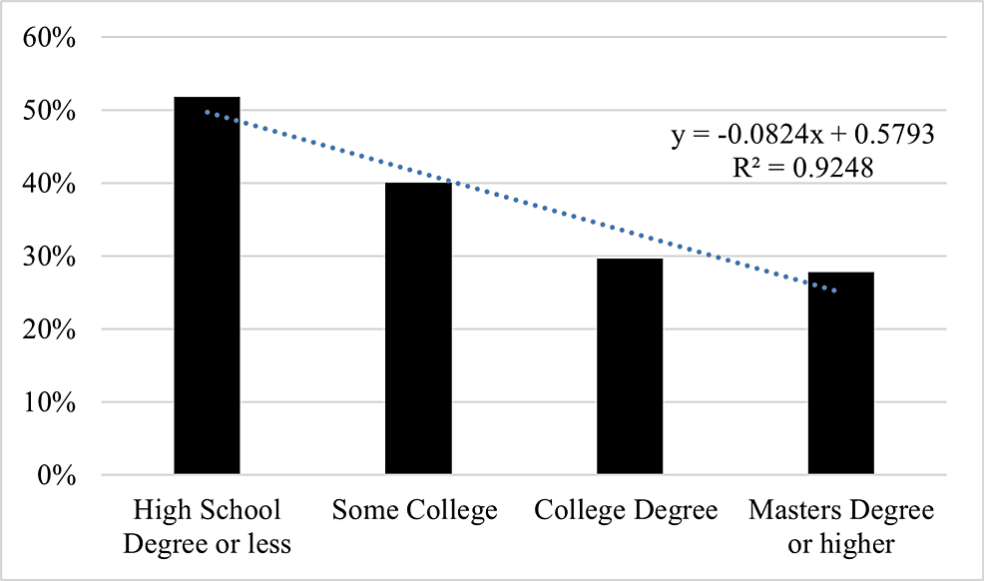
Education status of those who have never received ABA ABA: applied behavior analysis.

Participants documented their highest level of education as a high school diploma (r-ABA = 14.6%; n-ABA = 27.2%), some college or associate degree (r-ABA = 28.1%; n-ABA = 32.4%), or a bachelor’s degree or higher (r-ABA = 57.3%; n-ABA = 40.2%) (p = 0.003) (Table 1 and Figure 3). Logistic regression analysis was performed for both income (OR = 1.21, 95% CI = 1.08-1.37, p = 0.001) and education (OR = 1.19, 95% CI = 1.08-1.32) (p < 0.001).

Caregiver employment status

Participants reported having various levels of work responsibilities. Work activity of caregivers included those working full-time (r-ABA = 47.0%; n-ABA = 44.8%), part-time workers (r-ABA = 9.4%; n-ABA = 13.0%) to either full-time homemakers (r-ABA = 35.3%; n-ABA = 28.6%) or those unable to work due to disability (r-ABA = 2.3%; n-ABA = 8.4%) (p = 0.021) (Table 1).

Caregiver insurance

The majority of participants reported having either primary health insurance through an employer-sponsored plan (r-ABA = 37.1%; n-ABA = 35.0%), a government-sponsored plan (r-ABA = 54.4%; n-ABA = 58.6%), or coverage purchased directly from an insurance company (r-ABA = 3.9%; n-ABA = 2.5%) (p = 0.838). There was no statistical significance in ABA status based on insurance (Table 1).

Those who did not agree that proper treatment will improve the behavior of children with ASD were more likely to state they were not currently getting ABA (11.06 (2.61-46.89), p < 0.010) and were more likely to state they had to stop getting ABA because their insurance did not cover the therapy compared to those who did agree that treatment would improve behavior (OR = 7.05, 95% CI = 2.11-23.6, p < 0.010).

Never received ABA

Caregivers whose child was not receiving ABA cited the most common reasons their child has never received ABA include: long waitlist times (33.7%), they have heard bad things about ABA (13.7%), they feel overwhelmed and do not know where to start (12.6%), ABA was not covered by their insurance (10.3%), or there was no ABA in their area (10.3%). Additional reasonings included that the caregiver did not want the therapy at this time (10.3%), they have never heard of ABA therapy (9.1%), or did not have enough time to get their child to all their appointments (8.6%).

Factor analysis

Twelve questions were examined for factorability. Outliers were not an issue due to the fact all the questions exist on a scale from one to five. Eleven of these questions had at least one correlation with a magnitude of at least 0.3, which suggests reasonable factorability. The Kaiser-Meyer-Olkin measure of sampling adequacy was 0.82, above the recommended value of 0.60, and Bartlett’s test of sphericity was significant implying that these data are appropriate for factor analysis. It can be inferred that there is shared variance among the 12 questions due to the fact the final communality estimates were all above 0.30.

The question “I can always find the time to make all the phone calls I need to get my child into therapies” was eliminated at the start because it had large factor loadings on multiple factors. Three factors were selected because this produced a simple structure where each variable loaded highly only onto a single factor. The proportion of the variance explained by factor 1 was 55.09%, 32.42% for factor 2, and 12.49% for factor 3. The inter-factor correlation between factor 1 and factor 2 was 0.08, between factor 1 and factor 3 was 0.19, and between factor 2 and factor 3 was 0.41; due to the correlation between factor 2 and factor 3 being above 0.30, an oblique varimax rotation was done to account for this correlation (Table 2).

**Table 2:** Factor loadings in factor analysis

Questionnaire Items	Factor 1: Caregiver belief in treatment	Factor 2: Caregiver empowerment	Factor 3: Caregiver comfort level
With proper treatment, the DEVELOPMENT of children with autism will improve.	0.89	0.03	0.08
With proper treatment, the BEHAVIOR of children with autism will improve.	0.82	0.02	0.09
With proper treatment, children with autism can be more like children who do not have autism.	0.58	0.04	-0.06
The earlier a child gets TREATMENT for autism the more progress the child will make.	0.74	-0.05	0.08
I know which agencies, therapists, and doctors I need to contact to get my child the help he or she needs.	0.03	0.80	0.10
I know how to find the right services for my child with AUTISM.	-0.02	0.72	0.16
I find it easy to get the services my child needs to make progress.	0.03	0.67	-0.06
I know what to do if my child is NOT getting the right therapy services.	0.00	0.63	0.20
I am comfortable sending emails to get my child services.	0.06	-0.08	0.85
I am comfortable making phone calls to get my child services.	0.00	0.05	0.83
I take control to get the services that my child needs to make progress.	0.00	0.18	0.59

Factor 1: caregiver belief in treatment

Factor 1 measures the concept of caregiver belief in treatment and contains the variables child development information, behavior information, early treatment information, and treatment improvement information. Each question had a loading on this factor of at least 0.55 and loadings below 0.10 on all other factors. Factor 1 has a Cronbach's alpha of 0.811. The average of caregiver responses, on a five-point Likert scale, for factor 1 was greater in r-ABA (M = 4.20; SD = 0.72) than n-ABA (M = 4.06; SD =0.83) (t(467) = 1.91, p = 0.029) (Table 3).

**Table 3:** Caregiver belief, empowerment, and comfort level based on ABA status * = Statistically significant difference between subgroups defined as p < 0.050. ABA: applied behavior analysis.

Values	Received ABA	Never received ABA	p-value
Average of Factor 1: Caregiver belief in treatment	4.20	4.06	0.029*
Average of Factor 2: Caregiver empowerment	3.19	2.90	<0.001*
Average of Factor 3: Caregiver comfort level	4.35	4.03	<0.001*

Factor 2: caregiver empowerment

Factor 2 measures caregiver empowerment for getting treatment for their child and contains the variables of knowing who to contact, finding the right services, easy-to-get services, and knowing what to do if their child is not getting the right services. Each question had a loading on this factor of at least 0.60 and loadings below 0.20 on all other factors. Factor 2 has a Cronbach's alpha of 0.84. The average of caregiver responses, on a five-point Likert scale, for factor 2 was greater in r-ABA (M = 3.19; SD = 0.93) than in n-ABA (M = 2.90; SD = 1.00) (t(467) = 3.159; p < 0.001) (Table 3).

Factor 3: caregiver comfort level

Factor 3 measures caregiver comfort level and contains the variables comfortable sending emails, comfortable making calls, and taking control to get services for their child. Each question had a loading on this factor of at least 0.55 and loadings below 0.20 on all other factors. Factor 3 has a Cronbach's alpha of 0.82. The average of caregiver responses, on a five-point Likert scale, for factor 3 was greater in those who received ABA (M = 4.35; SD = 0.72) than those who never received ABA (M = 4.03; SD = 0.91) (t(466) = 4.289; p < 0.001) (Table 3).

## Discussion

There are many barriers to accessing adequate ABA and many children with ASD do not receive the services they need. By surveying caregivers, we identified factors preventing children with ASD from receiving ABA. The biggest barriers identified in this study were location, income, caregiver education, and time since the child’s ASD diagnosis as well as caregiver empowerment, belief in treatment, and comfort level accessing services.

Caregiver identity

Prior studies have shown that a gender imbalance among primary caregivers exists [19]. Our study echoes previous findings, as the majority of respondents were female between ages 30 and 44 years. Previous studies have also identified it as a barrier, showing African American children with ASD to be diagnosed at a later age and African American and Hispanic populations with decreased accessibility to ASD treatment when compared to Caucasian children [7,23-25]. Interestingly, race was not noted to be a significant ABA access barrier. We speculate the patient demographics of Nemours Children’s Hospital locations are contributory despite comparability to US Census racial rates [26].

Caregiver location

Survey data suggest location is contributory to ABA disparities. Data demonstrate an uneven number of BCBAs between US counties; more than half of all counties have none [27]. In our study, respondents who reported they did not know how to find the right services cited a lack of ABA in their area as a barrier to obtaining therapy for their child. Delaware had a higher rate of n-ABA children. Florida, New Jersey, and Pennsylvania, however, each had more children receiving services than not. Notably, the amount of BCBAs in each state is as follows: Delaware = 135, Florida = 7,306, New Jersey = 2,810, and Pennsylvania = 2315 [28]. The ratio of BCBA/10,000 people is as follows: Delaware = 1,311, Florida = 3,354, New Jersey = 3,041, and Pennsylvania = 1,793. While the reasons for these differences remain unclear, our study illuminates a need for further research to explore the impact of location on ABA initiation.

Caregiver income, education, and work activity

Cost is a barrier to receiving ABA [17]. Horlin et al. found that the median annual cost of having a child with ASD was $34,900, with nearly 90% of that cost due to loss of wages [17]. Our study showed that those with higher household incomes and higher educations were more likely to receive ABA for their child. This may be due to having better access to resources to facilitate treatment, such as time, transportation, or additional help outside the family. We found that of those who worked full-time, part-time, were homemakers, or were unemployed, a greater percentage of their children with ASD have received ABA. Those who were disabled were less likely to receive ABA. This may be due to the high cost of having a child with ASD even before paying for ABA; requiring that caregivers take on more hours to provide financially. Caregivers who work less or are unable to work may not be able to shoulder ABA costs. This may be due to lower income due to fewer hours or an inability to qualify for insurance benefits to allay treatment expenses. Lack of time may be another ABA barrier and suggests that those with more free time are better equipped to engage in intensive therapies for up to seven days a week.

Insurance

Over the past few decades, there has been an increase in both Medicaid and private insurance plans covering ABA. However, it is generally believed that caregivers with Medicaid or other government-sponsored plans have a longer wait time to access ABA. There are also differences in reimbursement rates that vary widely across plans [29]. Low reimbursement could deter qualified providers from contracting with particular insurance and, thus, limit available resources for families. Our study found that insurance status was not significant in caregivers securing ABA for their child. Additionally, our data suggest that insurance coverage, or lack thereof, seems to influence family perceptions regarding treatment benefits. Caregivers who did not agree that proper treatment would improve the behavior of children with ASD were more likely to state they were not currently getting ABA and were more likely to state they had to stop getting ABA because their insurance did not cover the therapy.

Time since ASD diagnosis

We noted a positive correlation between the number of children receiving ABA and the amount of time since ASD diagnosis. This is likely attributed to the well-recognized long wait times across the country [30].

Caregiver belief in treatment

We found caregiver belief in treatment was greater in those who received ABA than those who never received ABA. Caregivers whose child had never received ABA were less likely to believe that the earlier a child gets treatment for ASD the more progress the child will make and also less likely to believe that with proper treatment, behavior and development will improve. A previous study reported that 48% of families did not know anything about ASD before their child was diagnosed [6]. This emphasizes the need for providers to educate caregivers on the significant impact that these behavioral therapies, such as ABA, can have on their child’s behavior, development, and overall long-term outcomes.

Caregiver empowerment

There was a significant difference in caregivers’ level of empowerment seen in those who received ABA as compared to those who did not. Families who never received ABA were less likely to know how to find the right services, less likely to know what to do if their child was not getting the right services, and less likely to know which agencies, therapists, and doctors they needed to contact to get proper services for their child. However, caregiver empowerment regarding ABA has not yet been fully studied and further research is warranted.

Caregiver comfort level

Our data showed there was a significant difference in caregivers’ comfort levels between those who had received ABA and those who had not, with comfort levels greater in those who received ABA than those who never received ABA. Caregivers surveyed who had not received ABA for their children were less comfortable contacting child services through phone or email. This shows that caregiver comfort level is a barrier to receiving ABA. This underscores the need for greater caregiver education on ASD treatment options to promote greater comfort in utilizing the resources available.

Limitations

While this study successfully used a multisite, multistate children’s hospital system, the data collected for this study consisted of a survey, which inherently has limitations. Performing a survey analysis includes biases of each survey participant, subjectivity of answers, and the ability to recall specific barriers that they faced in the past. Additionally, this was a long 51-question survey, which could have created some fatigue during survey completion and could have led to participants not completing it in its entirety. Another limitation is that the survey was only provided in English and there may be language barriers that could contribute to accessing ABA that were not addressed in our study.

## Conclusions

A total of 540 caregivers of patients with ASD completed a 51-question survey to identify the barriers to children with ASD receiving ABA. Caregivers from Delaware noted a significant disparity in ABA accessibility. Caregivers who were more assertive had a positive outlook on treatment, and caregivers who had more knowledge about ABA were more likely to obtain it for their children. ABA accessibility is a multifactorial issue. Location, income, education of the caregiver, time since ASD diagnosis for the child, as well as caregiver empowerment, belief in treatment, and level of comfort in accessing services most likely contribute to children with ASD not receiving ABA. This emphasizes the need for providers to educate caregivers on the significant impact that these behavioral therapies, such as ABA, can have on their child’s behavior, development, and overall long-term outcomes.

## Appendices

Survey

1. Are you the primary caregiver of the child who has autism?

a. Yes

b. No

c. Other

2. If other, please explain: ______

3. What state do you currently live in?

a. Florida

b. Delaware

c. New Jersey

d. Pennsylvania

4. What is your relationship to the child who has autism?

a. Biological parent

b. Step-parent

c. Foster parent

d. Adoptive parent

e. Family member (grandparent/aunt/uncle)

f. Friend of the family

5. Has your child been formally evaluated and diagnosed with autism by a specialist? (Outside of your primary care provider)

a. Yes

b. No

c. Unsure

6. How long ago was your child diagnosed with autism?

a. Less than a month ago

b. 1-6 months ago

c. 7-12 months ago

d. 1-2 years ago

e. 3-5 years ago

f. Greater than 5 years ago

7. What is the primary health insurance for your child with autism?

a. Current or former employer or union (of the person completing this survey or another family member)

b. Insurance purchased directly from an insurance company (by the person completing this form or another family member)

c. Medicaid, Medical Assistance, CHIP, or any kind of government assistance plan for those with low incomes or a disability

d. TRICARE, VA, or other military health care

e. Indian Health Insurance

f. No health insurance

g. Other (please specify below)

8. If other, please specify the primary form of health insurance for your child with autism.

9. How many ADULTS are currently living in your house, including yourself?

a. 1

b. 2

c. 3

d. 4

e. 5

f. 6

g. 7

h. 8

i. 9

j. 10+

10. How many children UNDER the age of 18 years old are currently living in your house (including your child with autism)?

a. 1

b. 2

c. 3

d. 4

e. 5

f. 6

g. 7

h. 8

i. 9

j. 10+

11. I know how to find the right services for my child with AUTISM.

a. Strongly Agree

b. Agree

c. Neutral

d. Disagree

e. Strongly Disagree

12. I take control to get the services that my child needs to make progress.

a. Strongly Agree

b. Agree

c. Neutral

d. Disagree

e. Strongly Disagree

13. I know what to do if my child is NOT getting the right therapy services.

a. Strongly Agree

b. Agree

c. Neutral

d. Disagree

e. Strongly Disagree

14. I am comfortable making phone calls to get my child services.

a. Strongly Agree

b. Agree

c. Neutral

d. Disagree

e. Strongly Disagree

15. I am comfortable sending emails to get my child services.

a. Strongly Agree

b. Agree

c. Neutral

d. Disagree

e. Strongly Disagree

16. I like to connect with other parents on social media to find resources for my child.

a. Strongly Agree

b. Agree

c. Neutral

d. Disagree

e. Strongly Disagree

17. I can always find the time to make all the phone calls I need to get my child into therapy.

a. Strongly Agree

b. Agree

c. Neutral

d. Disagree

e. Strongly Disagree

18. I know which agencies, therapists, and doctors I need to contact to get my child the help he or she needs.

a. Strongly Agree

b. Agree

c. Neutral

d. Disagree

e. Strongly Disagree

19. I find it easy to get the services my child needs to make progress.

a. Strongly Agree

b. Agree

c. Neutral

d. Disagree

e. Strongly Disagree

20. If selected disagree or strongly disagree, please specify why: ______

21. The earlier a child gets TREATMENT for autism the more progress the child will make.

a. Strongly Agree

b. Agree

c. Neutral

d. Disagree

e. Strongly Disagree

22. With proper treatment, the BEHAVIOR of children with autism will improve.

a. Strongly Agree

b. Agree

c. Neutral

d. Disagree

e. Strongly Disagree

23. With proper treatment, the DEVELOPMENT of children with autism will improve.

a. Strongly Agree

b. Agree

c. Neutral

d. Disagree

e. Strongly Disagree

24. With proper treatment, children with autism can be more like children who do not have autism.

a. Strongly Agree

b. Agree

c. Neutral

d. Disagree

e. Strongly Disagree

25. Early treatment does NOT help children with autism.

a. Strongly Agree

b. Agree

c. Neutral

d. Disagree

e. Strongly Disagree

26. Has your child ever received ABA therapy?

a. Yes

b. No

27. If no, please select the one(s) that best match your experience:

a. Have not contacted anyone to get on the waitlist yet

b. On a waitlist, and waiting for services

c. Do not want the therapy at this time

d. My insurance doesn't cover the therapy

e. I have heard bad things about the therapy

f. I don't have transportation to get to and from my appointments

g. I don't have enough time to get the child to all appointments

h. Due to COVID-19, we are not comfortable getting therapy at this time

i. I have never heard of ABA therapy

j. There is no ABA in my area

k. Other services or therapies take priority over ABA

l. I am completely overwhelmed and don't know where to start

m. Other

28. If yes, how LONG has your child been in ABA therapy?

a. <1 month

b. 1-3 months

c. 4-6 months

d. 1 year

e. 2 years

f. 3 years

g. 4 years

h. 5 years

i. 6+ years

29. If yes, is your child currently in ABA therapy?

a. Yes

b. No

30. If no, why is your child no longer in ABA?

a. My child met all of the ABA goals

b. ABA provider went out of business

c. We moved to a new location and had to find a new provider

d. We did not like the ABA provider

e. We did not like ABA therapy for our child

f. ABA is not our priority right now

g. ABA providers do not speak our language

h. We had insurance issues that kept us from getting ABA

i. We changed insurance providers

j. My insurance doesn't cover ABA and we can't pay for it ourselves

k. We can't find a provider that matches our schedule

l. We had transportation issues that kept us from getting ABA

m. We were physically distancing due to COVID-19

n. Other

31. If no, do you plan to get your child back into ABA?

a. Yes

b. No

c. Unsure

32. How many HOURS a week of ABA does your child receive? (Enter a number between 0 and100)

33. Does your ABA provider TEACH YOU how to use ABA strategies in the home (aka parent training)?

a. Yes

b. No

34. If no, would you like them to teach you how to use ABA strategies in the home?

a. Yes

b. No

35. If yes, how OFTEN do they teach you ABA strategies (aka parent training)?

a. Multiple times a week

b. Once a week

c. Once a month

d. Every other month

e. A few times each year

36. Is your child's BEHAVIOR getting better in ABA (please select the one(s) that best match your experience)?

a. Yes, making gains very quickly

b. Yes, but it feels slow going at times

c. Yes, but my child only performs for the therapist and not for me

d. No, doesn't seem to be learning much at all

e. No, have not noticed any changes since my child started

37. Is your child making progress in LEARNING NEW SKILLS in ABA (please select the one(s) that best match your experience)?

a. Yes, making gains very quickly

b. Yes, but it feels slow going at times

c. Yes, but my child only performs for the therapist and not for me

d. No, doesn't seem to be learning much at all

e. No, have not noticed any changes since my child started

38. Since starting ABA, was there ever a time when your child was NOT getting ABA services?

a. Yes

b. No

39. If yes, why? (Select all that apply)

a. My child met all of the ABA goals

b. ABA provider went out of business

c. We moved to a new location and had to find a new provider

d. We did not like the ABA provider

e. We did not like ABA therapy for our child

f. ABA is not our priority right now

g. ABA providers do not speak our language

h. We had insurance issues that kept us from getting ABA

i. We changed insurance providers

j. My insurance doesn't cover ABA and we can't pay for it ourselves

k. We can't find a provider that matches our schedule

l. We had transportation issues that kept us from getting ABA

m. We were physically distancing due to COVID-19

n. Other

40. If yes, how long did your child go without ABA?

a. A few weeks

b. A few months

c. Many months

d. 1 year

e. 2 years

f. 3 or more years

41. What is your age?

a. 18-29 years

b. 30-44 years

c. 45-59 years

d. 60+ years

42. Which gender do you identify most with?

a. Male

b. Female

c. Other

d. Don't want to say

43. What is your most current marital status?

a. Single never married

b. Married or living with partner

c. Divorced

d. Separated

e. Widowed

44. Please specify your ethnicity.

a. Caucasian

b. African American

c. Latino or Hispanic

d. Asian

e. Native American

f. Native Hawaiian/Pacific Islander

g. Other/unknown

45. What is the HIGHEST level of school you completed?

a. Didn't finish high school

b. Didn't finish high school, but completed a technical/vocational program

c. Completed high school and a technical/vocational program

d. High school diploma or equivalency (GED)

e. Less than 2 years of college

f. Associate's degree (junior college) or equivalent

g. Bachelor's degree

h. Master's degree or other postgraduate training

i. Doctorate or other professional degree (PhD, EdD, DVM, MD, JD, DDS, etc.)

j. Other

46. If didn’t finish high school, what was the highest grade you completed? ______

47. Which of the following best describes your current daily activities and/or responsibilities?

a. Working full-time

b. Working part-time

c. Unemployed or laid off, not looking for work

d. Unemployed or laid off, looking for work

e. Homemaking or raising children full-time

f. Retired

g. Disabled (not working because of permanent or temporary disability)

48. What is your total household income in the past year, before taxes and other deductions?

a. Less than $5,000

b. $5,000 through $24,999

c. $25,000 through $49,999

d. $50,000 through $74,999

e. $75,000 through $99,999

f. $100,000 through $149,999

g. $150,000 +

h. Do not know

49. Is the home where you live:

a. OWNED by you or someone in the house (WITH a mortgage or loan)?

b. OWNED by you or someone in the house free and clear (WITHOUT a mortgage or loan)

c. RENTING a home or apartment

d. LIVING in a home/apartment without having to pay money or rent?

e. LIVING with family or friends (giving money for rent/expenses)

f. LIVING with family or friends (NOT giving money for rent/expenses)

g. Homeless

h. Other (please specify your living situation below)

50. If other, please specify your living situation. ______

51. Is this survey complete?

 a. Yes

 b. No

## References

[1] Hyman SL, Levy SE, Myers SM (2020). Identification, evaluation, and management of children with autism spectrum disorder. Pediatrics.

[2] Kanner L (1968). Autistic disturbances of affective contact. Acta Paedopsychiatr.

[3] Roane HS, Fisher WW, Carr JE (2016). Applied behavior analysis as treatment for autism spectrum disorder. J Pediatr.

[4] Maenner MJ, Warren Z, Williams AR (2023). Prevalence and characteristics of autism spectrum disorder among children aged 8 years - autism and developmental disabilities monitoring network, 11 sites, United States, 2020. MMWR Surveill Summ.

[5] Schelly D, González PJ, Solís PJ (2019). Barriers to an information effect on diagnostic disparities of autism spectrum disorder in young children. Health Serv Res Manag Epidemiol.

[6] Christensen DL, Bilder DA, Zahorodny W (2016). Prevalence and characteristics of autism spectrum disorder among 4-year-old children in the autism and developmental disabilities monitoring network. J Dev Behav Pediatr.

[7] Ennis-Cole D, Durodoye BA, Harris HL (2013). The impact of culture on autism diagnosis and treatment: considerations for counselors and other professionals. Fam J.

[8] Zwaigenbaum L, Bauman ML, Choueiri R (2015). Early intervention for children with autism spectrum disorder under 3 years of age: recommendations for practice and research. Pediatrics.

[9] Sacrey LA, Bennett JA, Zwaigenbaum L (2015). Early infant development and intervention for autism spectrum disorder. J Child Neurol.

[10] Fenske EC, Zalenski S, Krantz PJ, McClannahan LE (1985). Age at intervention and treatment outcome for autistic children in a comprehensive intervention program. Anal Interv Dev Disabil.

[11] Lovaas OI (1987). Behavioral treatment and normal educational and intellectual functioning in young autistic children. J Consult Clin Psychol.

[12] Tiura M, Kim J, Detmers D, Baldi H (2017). Predictors of longitudinal ABA treatment outcomes for children with autism: a growth curve analysis. Res Dev Disabil.

[13] Fisher WW, Luczynski KC, Blowers AP (2020). A randomized clinical trial of a virtual-training program for teaching applied-behavior-analysis skills to parents of children with autism spectrum disorder. J Appl Behav Anal.

[14] Freedman BH, Kalb LG, Zablotsky B, Stuart EA (2012). Relationship status among parents of children with autism spectrum disorders: a population-based study. J Autism Dev Disord.

[15] Hartley SL, Barker ET, Seltzer MM, Floyd F, Greenberg J, Orsmond G, Bolt D (2010). The relative risk and timing of divorce in families of children with an autism spectrum disorder. J Fam Psychol.

[16] Leaf JB, Leaf R, McEachin J (2016). Applied behavior analysis is a science and, therefore, progressive. J Autism Dev Disord.

[17] Horlin C, Falkmer M, Parsons R, Albrecht MA, Falkmer T (2014). The cost of autism spectrum disorders. PLoS One.

[18] Schelly D, Jiménez González P, Solís PJ (2018). Parental action and referral patterns in spatial clusters of childhood autism spectrum disorder. J Autism Dev Disord.

[19] Harrison AJ, Bradshaw LP, Naqvi NC, Paff ML, Campbell JM (2017). Development and psychometric evaluation of the autism stigma and knowledge questionnaire (ASK-Q). J Autism Dev Disord.

[20] Helton MR, Alber-Morgan SR (2018). Helping parents understand applied behavior analysis: creating a parent guide in 10 steps. Behav Anal Pract.

[21] Harris PA, Taylor R, Thielke R, Payne J, Gonzalez N, Conde JG (2009). Research electronic data capture (REDCap)--a metadata-driven methodology and workflow process for providing translational research informatics support. J Biomed Inform.

[22] Harris PA, Taylor R, Minor BL (2019). The REDCap consortium: building an international community of software platform partners. J Biomed Inform.

[23] Barnard-Brak L, Morales-Alemán MM, Tomeny K, McWilliam RA (2021). Rural and racial/ethnic differences in children receiving early intervention services. Fam Community Health.

[24] Maglione MA, Kadiyala S, Kress AM, Hastings JL, O'Hanlon CE (2016). TRICARE applied behavior analysis (ABA) benefit. RAND Health Q.

[25] Magaña S, Parish SL, Son E (2015). Have racial and ethnic disparities in the quality of health care relationships changed for children with developmental disabilities and ASD?. Am J Intellect Dev Disabil.

[26] (2023). U.S. Census Bureau. QuickFacts: United States. https://www.census.gov/quickfacts/fact/table/US/PST045221.

[27] Araiba S, Čolić M (2022). Preliminary practice recommendations for telehealth direct applied behavior analysis services with children with autism. [PREPRINT]. J Behav Educ.

[28] (2023). Behavior Analyst Certification Board. Region-specific certificant data. https://www.bacb.com/services/o.php?page=101134.

[29] Maglione M, Kadiyala S, Kress A, Hastings JL, O'Hanlon CE (2017). TRICARE applied behavior analysis (ABA) benefit: comparison with Medicaid and commercial benefits. Rand Health Q.

[30] (2023). Prevalence of delays to receiving behavioral therapy in Michigan: a summary of survey data. https://autismallianceofmichigan.org/contributed-by-adam-m-briggs-phd-bcba-d-lba-andrea-m-peterson-ma-bcba-lba-eastern-michigan-university/.

